# Can mindfulness mitigate the energy-depleting process and increase job resources to prevent burnout? A study on the mindfulness trait in the school context

**DOI:** 10.1371/journal.pone.0214935

**Published:** 2019-04-04

**Authors:** Gloria Guidetti, Sara Viotti, Rosa Badagliacca, Lara Colombo, Daniela Converso

**Affiliations:** Dipartimento di Psicologia, Università degli Studi di Torino, Turin, Italy; University o f Genoa, ITALY

## Abstract

**Background:**

Past studies in the teaching context provided evidence of the role of mindfulness-based intervention in improving occupational wellbeing. This study aims to increase the extant knowledge by testing the mechanism that links teachers’ mindfulness at work to occupational wellbeing. Rooted in the job demand–resource model, the mindfulness trait is conceptualized as a personal resource that has the ability to impact and interact with job demands and resources, specifically workload stress appraisal and perceived meaningfulness of work, in affecting teachers’ burnout.

**Methods:**

A sample of primary, middle, and secondary school teachers (N = 605) completed a questionnaire that aimed to assess teachers’ mindfulness trait and the measures of the quality of occupational life in the school context. Confirmatory factor analysis (CFA) was conducted to test the model fit indices; further analyses were performed to test the hypotheses about mediation and moderation effects.

**Results:**

The CFA showed good model fit indices. Further analyses highlighted that teachers’ mindfulness is negatively associated with workload stress appraisal and that positively influenced work meaning, in turn mediating the relationship between mindfulness and burnout. Finally, mindfulness moderated the effect of workload stress appraisal on burnout.

**Conclusions:**

Rooted in the job demand–resource model, this study emphasizes an underrepresented personal resource, that is, the mindfulness trait at work, and the links that favor its impact on burnout. Practical and future research implications are also discussed.

## Introduction

### Mindfulness at work

The concept of mindfulness underlines the ability to pay attention to both internal and external stimuli in the present moment and observe them without judgmental attitudes or cognitive distortions derived from heuristics [1]. It has been well defined its role in promoting wellbeing in both clinical and non-clinical samples, especially involving studies on the effectiveness of mindfulness-based interventions (MBIs, e.g., mindfulness-based stress reduction) [2] [3].

Some of the studies in the field of occupational health psychology began to evaluate mindfulness as a trait, called workplace mindfulness [4], which is a stable or a dispositional personal characteristic. Different from other psychological resources, such as self-efficacy, locus of control, or self-esteem, which have been the subjects of a wide research stream (e.g., [5]), the role of mindfulness as a psychological resource that is able to sustain occupational health has not been well examined to date. Recently, increased amount of studies has emphasized the importance of examining the mechanism linking mindfulness to wellbeing outcomes. For example, research has provided evidence that when a job task requires emotional regulation abilities, being mindful decreases the tendency to adopt surface acting strategies [6]. Other studies have indicated that the mindfulness trait at work is linked to work engagement via accrued personal resources (i.e., positive job-related affect) [7], and authentic functioning [8] which is an open and non-defensive way of interacting with oneself and others. Samios [9] reports that in a sample of mental health workers, mindfulness exerted an influence on depression alleviation, job satisfaction, and positive affect through the role of compassion satisfaction, which is the pleasure derived from taking care of patients.

All these results could be explained by the mechanism of reperceiving [10], that is, the ability to decouple the self from the event, favoring a positive reappraisal of potentially threatening stimuli derived from the external environment or from internal experiences. These studies have also proven mindfulness as a resource that helps people stay in touch with their true selves, needs, and values, which coincides with self-determination [11]. Based on the self-determination theory [11], Schultz et al. [12] showed that the mindfulness trait buffers the impact of an unsupportive work environment on the frustration from unfulfilled basic psychological needs.

Within the teaching profession the interest in the role of mindfulness has generated a large number of training-based experiences aimed to reduce stress and burnout, as well as its physiological, psychological, and organizational costs [13] [14] [15] [16]. In a recent systematic review of MBIs’ effects on stress, emotion regulation, and self-efficacy [17], its most significant finding relates MBIs to a decrease in perceived stress and an improvement in teachers’ mindfulness. Nevertheless, these studies do not provide clear evidence of the mechanism through which the benefits of participating in MBIs have an impact on reducing distress, such as burnout.

Because mindfulness concerns the ability to maintain awareness and attention at the present moment, with non-judgmental attitudes toward oneself and others, it is important to analyze the degree to which teachers are able to adapt these psychological functions to their daily work in the school context. As explained by Frank, Jennings, and Greenberg [18], these abilities are self-regulated behaviors that allow teachers to be more aware of the ongoing occurrences in the whole classroom, paying close attention to students’ needs and behaviors without being distracted by other work-related problems or acting using dysfunctional cognitive habits in front of stressful situations.

Rooted in the Job Demand–Resource model (JD-R) [19] [20], this study has the aim to enhance the extant knowledge about the role of mindfulness as a personal resource at work, to which scarce attention has been paid. Specifically regarding the teaching profession, which is widely considered a stressful occupation [21] [22], it is important to analyze how the mindfulness trait at work could impact one of the most relevant stress-related phenomena, that is, burnout, “a prolonged response to chronic emotional and interpersonal stressors on the job” ([23], p. 397). The two core dimensions of burnout are (1) emotional exhaustion, which refers to feelings of being overextended and depleted of one’s emotional and physical resources, and (2) cynicism (or depersonalization), which pertains to negative, callous, or excessively detached responses to various aspects of the job [23] [24]. Results of the present study thus may provide information about more targeted MBIs for teachers, indicating how mindfulness could prevent the onset of burnout.

### Role of mindfulness in the JD–R model

The JD–R model [20] is a well-established theoretical model in the field of occupational health psychology that explains how work conditions, categorized into job demands and resources, could affect employees. Job demands are the physical, psychological, or socio-organizational aspects of the work whose energy-depleting process induces people to experience energy loss and fatigue, leading to stress, illness, and burnout. On the other side, a motivational process is sustained by the presence of job resources, which refers to those physical, psychological, social, or organizational aspects of the job that reduce job demands and the associated physiological and psychological costs, while stimulating personal growth, learning, and development [25] [26] [27]. Moreover, as it is contented by the Conservation of Resource theory [28][29] even the lack of job resources represents a condition that could negatively impact wellbeing through the onset of stress and burnout.

A subsequent development of the JD–R model [19] introduced the role of psychological resources that are the “personal factors or aspects of the self that are generally associated with resiliency and refer to the ability to control and impact one’s environment successfully” ([30], p.49), that lead to more positive self-evaluations and stimulate optimal functioning [31] [32]. Within the JD-R model, personal resources, other than directly impact on wellbeing outcomes, are conceptualized as antecedents of either job demands or resources, such that they could influence the way in which employees perceive their work environment. Moreover, they can exert a buffer effect; in other words, they can moderate or influence the relations between job characteristics and wellbeing outcomes. Such that, people who have more personal resources can deal more effectively with job demands and, integrating the assumption of the COR theory [28][29] personal resources can compensate with the lack of job resources in affecting its negative consequences.

Regarding job demands, one of the most relevant sources of distress for teachers is excessive workload [33] [34] [35] [36] [37]. As pointed out by Van Droogenbroeck et al. [22], the multiple changes that the educational system has undergone over the last decade could be referred to as the processes that have intensified the teaching profession. This means that teachers face an increasing number of non-teaching-related demands, such as performing administrative work, participating in meetings, and keeping updated with new educational and pedagogical approaches that often reflect in the double bind of being adherent to teaching task and to be committed, at the same time, to the pedagogical mission of students’ education. Moreover, teachers have to interact with an increasing number of students per classroom, to whom they bear greater responsibilities.

Nevertheless, as stated by the transactional stress theory [38], rather than the sources of stress *per se*, it is important to observe the way in which people appraise potentially stressful situations. In this vein, experienced distress occurs every time a person perceives the situation as too demanding or threatening, thus exceeding one’s resources to deal with it. As shown in the study of Gomes et al. [37], the negative cognitive appraisal of demanding work characteristics or threat perception performs a central role in the onset of subsequent negative outcomes for teachers, thus mediating the relation between workload and burnout. The differentiation between sources of stress, such as workload, and people’s cognitive appraisal of them, is a key to the study on mindfulness. As stated earlier, mindfulness involves a greater capacity to reperceive external and internal stimuli, focusing on the present moment instead of activating maladaptive cognitive schemas. In this way, Weinstein, Brown, and Ryan [39] showed that dispositional mindfulness alters the stress process, lessening negative appraisals of stress and in turn favoring more adaptive cognitive strategies.

Although the cited literature indicates how mindfulness could alter the negative appraisal of stress, no studies to date have explored this relationship, considering the role of mindfulness as a psychological resource in the JD–R model [19]. For example, few studies showed how mindfulness enhances one’s ability to deal with job demands [40] [4], but they consider neither the impact of mindfulness on the negative appraisal of stress-inducing job demands nor the relationship with job resources.

In addition to altering the negative appraisal of stress, as previously stated, mindfulness develops a person’s capacity to be more self-determined, focused on the current work tasks, and to act in accordance with one’s true self and values [8] [9] [12]. One of the job resources that could be closely linked to the expression of a person’s own values and self-determined behaviors at work is the perception about one’s meaning of work. The meaning of work has been defined as an individual’s subjective sense of one’s work situation, expressed through job-related activities that are congruent with one’s personal values [41] [42]. Regarding the teaching profession, perceived meaningfulness of work represents an important source of intrinsic reward [43], where teaching constitutes a way of expressing oneself as a human being by working with and influencing students [44]. Previous studies have analyzed antecedents [45] and outcomes of meaningfulness of work [46] [47] [48] as a fundamental job resource. However, to date, no studies have examined the role of personal resources, such as mindfulness, in relation to meaningful work, and there are scarce findings about this resource for the teaching profession.

Finally, none of the previous studies used context-specific mindfulness measures. This tenet is of further importance, as the JD–R model [20] implies that job and personal resources should be typical of the specific occupation.

Given the above literature review, it is argued here that mindfulness plays a central role in affecting, on one hand, the workload stress appraisal that constitutes the energy-depleting process leading to burnout, and on the other hand, in fostering meaningfulness of work of teachers, as it favors a better alignment with self-determined behaviors and the focus on the core characteristics of the teaching process, thus preventing from the onset of burnout.

**Fig 1:**
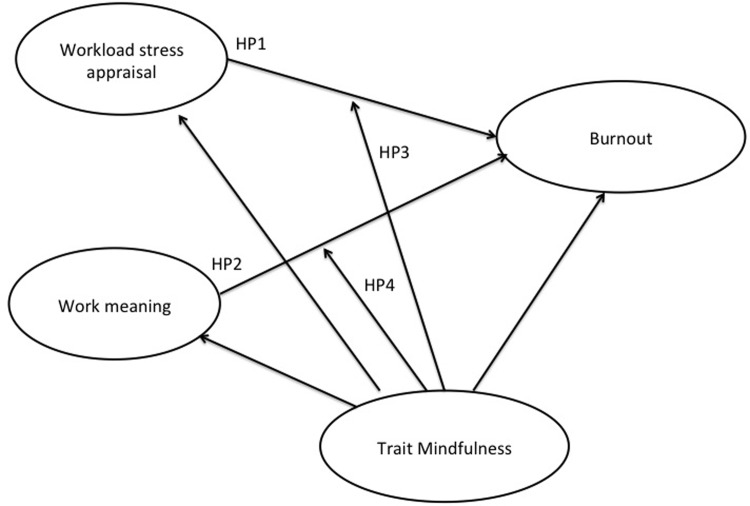
Model of hypothesis tested.

The following hypotheses are formulated (Fig 1) regarding indirect path linking mindfulness to burnout:

HP1. Workload stress appraisal mediates the relationship between mindfulness and both emotional exhaustion and depersonalization; that is mindfulness exerts its negative effect on burnout, lessening the negative appraisal of stress.

HP2. Teachers’ perceived meaningfulness of work mediates the relationship between mindfulness and both emotional exhaustion and depersonalization; that is, mindfulness exerts its negative effect on burnout, fostering the perception about meaningful work.

Moreover, given the double role played by personal resources within the JD-R model [19] [20], it is analyzed the buffer effect of mindfulness on both workload stress appraisal and meaningfulness of work:

HP3. Mindfulness moderates the effects of the negative appraisal of the workload on both emotional exhaustion and depersonalization; as mindfulness increases, the positive relationship between workload stress appraisal and burnout decreases, or stop being significant.

HP4. Mindfulness moderates the effects of perceived meaningfulness of work on both emotional exhaustion and depersonalization. Specifically as the lack of job resources could increase levels of burnout, personal resources can compensate them. Integrating COR theory assumption [28] [29] within the JD-R model [19] [20], it could be hypothesized that the more mindful people experience, lesser are the levels of burnout when meaningfulness of work is low, compared to those with high levels of meaningfulness of work.

## Materials and methods

### Data collection and participants

A cross-sectional design was used for data collection by means of a self-reported questionnaire administered from December 2016 to February 2017. The data was collected under a research program that aimed to assess the quality of teachers’ work life in a metropolitan area in northern Italy.

Research objectives, schedule and content of the questionnaire were approved by the Principals and teachers’ representative of the sixteen educational institutes involved in the research project: 66 Martiri (Turin), A. Frank (Turin), Regio Parco (Turin), L. Da Vinci, (Turin), Salvemini (Turin), Cherasco, (Cuneo), Diano D’Alba (Cuneo), Quartiere Moretta (Cuneo), Sommariva (Cuneo), Bodoni (Turin), Curie-Levi (Turin), Curie-Vittorini (Turin), A. Einstein (Turin), S. Grandis (Cuneo), Govone (Cuneo), Licei Giolitti-Gandino (Cuneo).

The participants volunteered for the research without receiving any reward, signed the informed consent forms, and agreed to anonymously complete the questionnaire. The research conforms to the Declaration of Helsinki of 1995 (as revised in Edinburgh 2000), and all ethical guidelines were followed, as required for conducting human research, including adherence to the legal requirements of the country under study. An additional ethical approval was not required since no treatment was involved, including medical, invasive diagnostics, or procedures causing psychological or social discomfort for the participants.

At their own convenience, the teachers returned the completed questionnaires in sealed boxes. Overall, the response rate was 49.75% (985 of the 1,980 questionnaires were returned). The majority of the participants were women (792, 80.4%). The sample had a mean (M) age of 45.7 years (standard deviation [SD] = 9.67; minimum = 23, maximum = 65). Based on the grade level, the sample consisted of 407 (41,3%) primary, 199 (20.2%) middle, and 379 (38.5%) secondary school teachers.

### Measures

Teachers’ mindfulness at work was measured using the intrapersonal mindfulness dimension of the Mindfulness in Teaching Scale [18]. Consisting of nine items on a five-point Likert scale ranging from 1 = totally false to 5 = totally true, it aimed to measure teachers’ non-judgmental and non-reactive abilities during their daily teaching activities that could help them mindfully observe their emotional reactivity and ability to pay attention at the present moment (e.g., “When I am in the classroom, I have difficulty in staying focused on what is currently happening.”). The scale had satisfactory reliability (α = 0.84). The index was calculated after reversing all the item scores, and the M and the SD of the whole sample were 39.65 and 4.25, respectively.

The teachers’ workload appraisal was measured using three items of an adaptation [49] of the Teacher Stress Inventory proposed by Klassen [50]. The items were presented with the prompt question: “How stressful are these aspects of your work for you as a teacher?” (e.g., “having extra duties/responsibilities,” “having a large class size,” “being responsible for students’ achievement”). The responses were given on a nine-point Likert scale ranging from 1 = not stressful at all to 9 = completely stressful. The scale had satisfactory reliability (α = 0.71). The M and the SD of the whole sample were 16.82 and 5.58, respectively.

Perceived meaningfulness of work was measured using three items of the Copenhagen Psychosocial Questionnaire (COPSOQ) [51] (e.g., “As a teacher, I think that my work is meaningful.”). The responses were given on a four-point scale ranging from 1 = totally disagree to 4 = totally agree. The M and the SD were 10.95 and 1.41, respectively. The scale had good reliability (α = 0.84).

Burnout was measured using the Maslach Burnout Inventory education survey [52] [53] with eight items about emotional exhaustion (e.g., “I feel emotionally drained from my work.”) (M = 14.80, SD = 10.02, α = 0.88) and four items about depersonalization (e.g., “I happen to treat students as impersonal objects.”) (M = 1.94, SD = 3.20, α = 0.68). The responses were given on a seven-point Likert scale (from 0 = never to 6 = every day).

All the scales, except for emotional exhaustion and depersonalization, were adapted to Italian using back translation [54].

### Data analysis strategies

The data was analyzed using the IBM Statistical Package for the Social Sciences (SPSS 25) and Mplus 7.3. The descriptive statistics included correlations among the major study variables.

First, to ascertain the adequacy of the psychometric properties and the reliability of the multi-item scales (i.e., teacher mindfulness at work, workload stress appraisal, perceived meaningfulness of work, emotional exhaustion, and depersonalization), a series of confirmatory factor analyses (CFAs) was performed to determine whether the best structure for the data was the hypothesized one. Five competing models, which included from one to five factors, were compared. The robust maximum likelihood was used as an estimation method. The model’s goodness of fit was assessed using the ratio of χ2 to the degrees of freedom (df), the comparative fit index (CFI), the Tucker-Lewis index (TLI), the standardized root mean square residual (SRMR), and the root mean square error of approximation (RMSEA). According to Kline [55], an χ2/df ratio of 3 or less indicates a good model fit, and less than 2 indicates an excellent model fit. For the TLI and the CFI, values higher than 0.90 are considered indicators of a good model fit [56]. An SRMR value of less than 0.08 indicates a good fit [57], and an RMSEA value lower than 0.09 indicates an acceptable model fit [58]. Additionally, the Akaike Information Criterion (AIC) and the Bayes Information Criterion (BIC) were used to compare the alternative (non-nested) measurement models [55]. For the AIC and the BIC, smaller values are indicative of better fitting models.

Second, to test the hypotheses, a series of mediation and moderation analyses was conducted separately for both emotional exhaustion and depersonalization, using the PROCESS macro for SPSS 25 [59]. The macro allowed simple mediation analyses for HP1 and HP2 and moderation analyses to test HP3 and HP4.

For the mediation analyses, the bootstrap sampling procedure was used to generate a 95% confidence interval around the indirect effect to test for its significance. The bootstrap confidence intervals were constructed using 5,000 samples.

All the analyses (CFA, mediation and moderation) were implemented using listwise deletion for missing data.

## Results

### Descriptive analyses and measurement model

Table 1 reports the correlations among the study variables. The teachers’ mindfulness trait correlated with all the other variables in the expected direction.

**Table 1:** Correlations among study variables.

	1	2	3	4	5	6
1. (Trait mindfulness)						
2. (Workload stress appraisal)	-.158^**^					
3. (Work Meaning)	.181^**^	.036				
4. (Emotional Exhaustion)	-.323^**^	.451^**^	-.156^**^			
5. (Depersonalization)	-.429^**^	-.180^**^	-.170^**^	.440^**^		
6. Age	NS	NS	-.136.^**^	.174^**^	NS	
7. Gender	-.128^**^	NS	NS	NS	.216^**^	NS

** p < .001; Male = 1, Female = 0

Table 2 presents the fit indices of the CFA. The hypothesized measurement model, consisting of five correlated latent factors, was the only model showing a good fit. The AIC and the BIC confirmed that this five-factor model fit the data significantly better than the alternative models that included one to four factors.

**Table 2:** Goodness-of-fit indexes—confirmatory factor analyses (CFA) of the constructs considered in the study.

	χ^2^(df)	χ^2^/df	CFI	TLI	SMRM	RMSEA	AIC BIC
M1 (5 constructs loaded on 1 factor)	3899.92(324)	12.03	.52	.48	.11	.10 (CI .103-.109)	71289.144 71684.705
M2 (4 constructs loaded on 1 factor vs trait mindfulness	2446.802(323)	7.57	.72	.69	.10	.08 (CI .079-.069)	69496.099 69896.543
M3 (Work meaning, emotional exhaustion and depersonalization loading on 1 factor, vs trait mindfulness and workload stress appraisal)	2163.337(321)	6.73	.75	.73	.08	.07 (CI .07 - .08)	69553.999 69553.999
M4 (Emotional exhaustion and depersonalization loaded on 1 factor vs trait mindfulness, workload stress appraisal and work meaning)	1271.756(318)	3.99	.87	.86	.06	.05 (CI .052 - .059)	68037.311 68462.173
M5 (Five factor model)	934.201(314)	2.97	.91	.90	.04	.04 (CI .042 - .048)	67622.008 68066.403

N. of cases of the model after listwise deletion: 916

### Results of hypothesis tests

To test the simple mediation analysis, four distinct models were calculated to determine the indirect effect of workload stress appraisal and perceived meaningful of work, respectively, between mindfulness and both emotional exhaustion and depersonalization.

The simple mediation results are presented in Tables 3–6.

**Table 3:** Simple mediation results of workload stress appraisal between mindfulness and emotional exhaustion.

Predictor	Model 1 Workload stress appraisal		Model 2 Emotional exhaustion B(SE)	
Control variables	**B (SE)**	**p**	**B (SE)**	**p**
Age	.05 (.02)	.00		
Gender	-.78 (.58)	.18		
Grade level: primary school	-.16 (.49)	.73		
Grade level: middle school	-1.52 (.60)	.01		
Indipendent variable				
Trait mindfulness	-.21(.05) path a	.00	-.77 (.08) path c	.00
			-.62 (.08) path c^1^	.00
Workload stress appraisal			.69 (.06) path b	.00
R^2^ = .29				
**Bootstrap indirect effect on Emotional exhaustion (through workload stress appraisal)**	**B**		**LL 95% CI**	**UL 95% CI**
Trait mindfulness	-.15 path (axb)		-.24	-.06

**Notes**: path a = effect of trait mindfulness on teachers’ workload stress appraisal. b = effect of teachers’ workload stress appraisal on emotional exhaustion. c = total effect of trait mindfulness on emotional exhaustion. c1 = direct effect of trait mindfulness on emotional exhaustion. Indirect effect (axb) of trait mindfulness on emotional exhaustion, through workload stress appraisal.

**Abbreviations**: LL, lower level; UL, upper level.

N. of cases of the model after listwise deletion: 606

**Table 4:** Simple mediation results of workload stress apprasial between mindfulness and depersonalization.

Predictor	Model 1 Workload stress appraisal		Model 2 Depersonalization	
Control variables	**B (SE)**	**p**	**B (SE)**	**p**
Age	.05 (.02)	.01	.00 (.01)	.94
Gender	-.64 (.57)	.26	1.10 (.26)	.00
Grade level: primary school	-.10 (.48)	.82	-.66 (.22)	.00
Grade level: middle school	-1.75 (.58)	.00	-.71 (.27)	.00
Indipendent variable				
Trait mindfulness	-.21 (.05) path a	.00	-.25 (.02) path c	.00
			-.24 (.02) path c^1^	.00
Workload stress appraisal			.06 (.01) path b	.00
R^2^ = .23				
**Bootstrap indirect effect on Depersonalization (through workload stress appraisal)**	**B**		**LL 95% CI**	**UL 95% CI**
Trait mindfulness	.01 path axb		-.02	-.00

**Notes**: path a = effect of trait mindfulness on teachers’ workload stress appraisal. b = effect of teachers’ workload stress appraisal on depersonalization. c = total effect of trait mindfulness on depersonalization. c1 = direct effect of trait mindfulness on depersonalization. Indirect effect (axb) of trait mindfulness on depersonalization, through workload stress appraisal.

**Abbreviations**: LL, lower level; UL, upper level.

N. of cases after listwise deletion: 627

**Table 5:** Simple mediation results of work meaning between mindfulness and emotional exhaustion.

Predictor	Model 1 Work meaning		Model 2 Emotional exhaustion	
Control variables	B (SE)	p	B (SE)	p
Age	-.015 (.005)	.00	.17(.03)	.00
Gender	-.02 (.14)	.88	-1.53(.97)	.11
Grade level: primary school	.43 (.11)	.00	-.68(.82)	.40
Grade level: middle school	.12 (.14)	.37	-2.13(1.01)	.03
Indipendent variable				
Trait mindfulness	.05 (.01) path a	.00	-.79 (.08) path c	.00
			-.76 (.08) path c^1^	.00
Work meaning			-.61 (.27) path b	.02
R^2^ = .16				
**Bootstrap indirect effect on Emotional exhaustion (through work meaning)**	**B**		**LL 95% CI**	**UL 95% CI**
Trait mindfulness	-.03 path (axb)		-.08	-.00

**Notes**: path a = effect of trait mindfulness on teachers’ work meaning. b = effect of teachers’ work meaning on emotional exhaustion. c = total effect of trait mindfulness on emotional exhaustion. c1 = direct effect of trait mindfulness on emotional exhaustion. Indirect effect (axb) of trait mindfulness on emotional exhaustion, through work meaning.

**Abbreviations**: LL, lower level; UL, upper level.

N. of cases after listwise deletion: 612

**Table 6:** Simple mediation results of work meaning between mindfulness and depersonalization.

Predictor	Model 1 Work meaning		Model 2 Depersonalization	
Control variables	B (SE)	p	B (SE)	p
Age	-.01 (.00)	.00	.00 (.01)	.87
Gender	-.03 (.14)	.82	1.16 (.27)	.00
Grade level: primary school	.49 (.11)	.00	-.69 (.22)	.00
Grade level: middle school	.19 (.14)	.17	-.74 (.27)	.00
Indipendent variable				
Trait mindfulness	.05(.01) path a	.00	-.27 (.02) path c	.00
			-.25 (.02) path c^1^	.00
Work meaning			-.23 (.02) path b	.00
R^2^ = .23				
**Bootstrap indirect effect on Depersonalization (through work meaning)**	**B**		**LL 95% CI**	**UL 95% CI**
Trait mindfulness	-.005 path (axb)		-.03	-.00

**Notes**: path a = effect of trait mindfulness on teachers’ work meaning. b = effect of teachers’ work meaning on depersonalization. c = total effect of trait mindfulness on depersonalization. c1 = direct effect of trait mindfulness on depersonalization. Indirect effect (axb) of trait mindfulness on depersonalization, through work meaning.

**Abbreviations**: LL, lower level; UL, upper level.

N. of cases after listwise deletion: 636

First of all, trait mindfulness is significantly and negatively associated to both emotional exhaustion and depersonalization. Tables 3 and 4 show how the mindfulness trait is negatively and significantly related to workload stress appraisal (Path a, Tables 3 and 4), which in turn is positively related to both emotional exhaustion (Path b, Table 3) and depersonalization (Path b, Table 4). Additionally, a significant indirect effect emerges, confirming HP1; path c1 (Tables 3 and 4) is significant, highlighting a partial mediation effect, as the effect of mindfulness on the outcome variables is still significant even in the presence of the mediation variable [60].

The analysis show that the results are consistent with the HP 2, as mindfulness significantly and positively relates to perceived meaningfulness of work (Path a, Tables 5 and 6), which in turn negatively relates to both emotional exhaustion (Path b, Table 5) and depersonalization (Path b, Table 6). Furthermore, significant indirect effects emerge, confirming HP2; mindfulness exerts its effect on both emotional exhaustion (path axb, Table 5) and depersonalization (Path axb, Table 6) through the mediation role of work meaning. Supporting the findings of Baron and Kenny [60], there is a partial mediation effect of perceived meaningfulness of work between mindfulness and both emotional exhaustion and depersonalization.

To test HP3 and HP4, a series of simple moderation analyses was performed. Tables 7 and 8 show only the results regarding the significant interaction between mindfulness and workload stress appraisal because no significant interaction effect emerges between mindfulness and perceived meaningfulness of work. These results confirm HP3 but not HP4.

**Table 7:** Simple moderation between mindfulness and workload stress appraisal on emotional exhaustion.

Predictors	B (SE)	p
Age	.13 (.03)	.00
Gender	-.96 (.91)	.28
Grade level: primay school	-.34 (.77)	.65
Grade level: middle school	-.96 (.84)	.25
Trait mindfulness	-.62 (.09)	.00
Workload stress appraisal	.70 (.05)	.00
Mindfulness x Workload stress appraisal	-.02 (.01)	.01

R^2^ = .29

N. of cases after listwise deletion: 607

**Table 8:** Simple moderation between mindfulness and workload stress appraisal on depersonalization.

Predictors	B (SE)	p
Age	-.00 (.13)	.86
Gender	1.19 (.34)	.00
Grade level: primay school	-.59 (.21)	.00
Grade level: middle school	-.57 (.26)	.02
Trait mindfulness	-.24 (.03)	.00
Workload stress appraisal	.07 (.01)	.00
Mindfulness x Workload stress appraisal	-.01 (.00)	.03

R^2^ = .24

N. of cases after listwise deletion: 628

**Fig 2:**
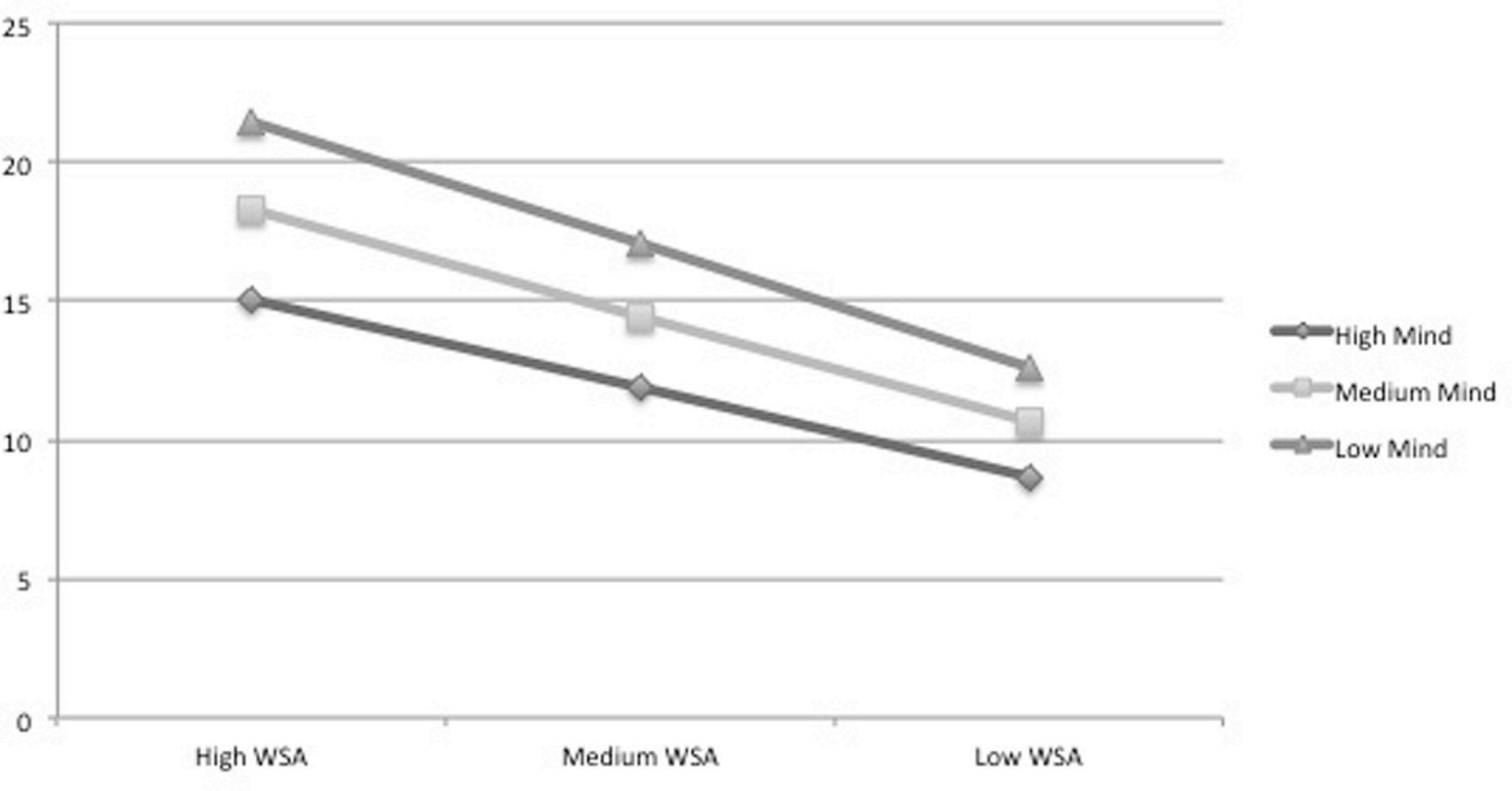
Emotional exhaustion at levels of trait mindfulness and workload stress appraisal. Note: WSA: Workload stress appraisal; Mind: Mindfulness.

**Fig 3:**
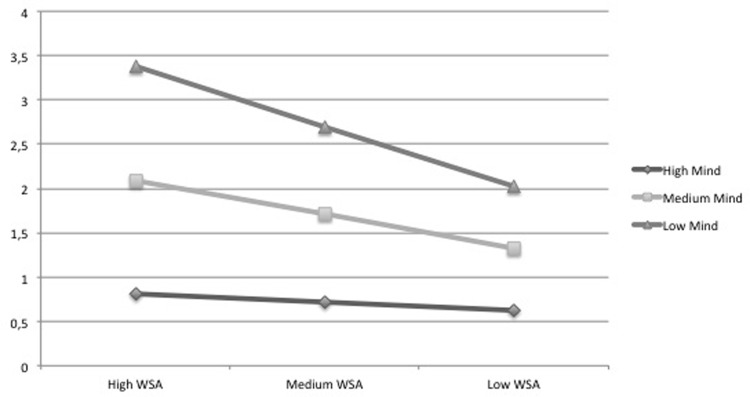
Depersonalization at levels of trait mindfulness and workload stress appraisal. Note: WSA: Workload stress appraisal; Mind: Mindfulness.

As emerging from the moderation analyses, conditional direct effects of workload stress appraisal on emotional exhaustion are significantly stronger at lower (-1 SD) and medium levels of mindfulness (M = 0) compared with higher levels (+1 SD) (Fig 2), while the conditional direct effects of workload stress appraisal on depersonalization are positive and significant only at lower (-1 SD) and medium (M = 0) levels of mindfulness, not at higher levels (+1 SD) (Fig 3). These results are in line with HP3; as mindfulness increases, the effect of workload stress appraisal on emotional exhaustion decreases, and it stops being significant in its relationship with depersonalization.

All the models have been controlled for potentially confounding variables, such as gender, age, and grade level (primary, middle, and secondary school teachers). All the results remain stable even without control variables.

## Discussion

The aim of this study was to increase the extant knowledge about the role of trait mindfulness as a psychological resource. Specifically, we focused our examination on the teaching profession, using a context-specific measure of mindfulness at work. Consistent with the JD-R model [19] [20], and with past studies on the role of mindfulness [8] [9] [11] [39], the study examined if trait mindfulness for teachers could impact on the perception of the workload stress appraisal and on a specific job resource, that is perceived meaningfulness of work and, at the same time, if mindfulness at work is a resource able to modify the impact that these job characteristics have on burnout.

Consistent with previous findings [39], results from this study have evidenced how mindfulness lessens the negative stress appraisal process that leads to experience burnout symptoms. This result widens previous studies that linked the cognitive appraisal of teacher stress to burnout [37], giving relevance to an underrepresented psychological resource able to lessen the effect exerted from negative appraisal. Extant literature conducted within the occupational health stream, has indeed taken into account the relations that mindfulness has with job demands, which, differently from cognitive appraisal, cannot be often directly modified by the employees [4] [6] [37] [40]. Therefore, the results of the present study give further importance to mindfulness as a resource able to modify the impact of negative cognitive schemas on the onset of burnout, and how these elements could be integrated within the JD-R model.

Moreover, our hypothesis on the role of mindfulness in fostering the perception of meaningful work have been confirmed. Consistent with previous studies [8] [9] [10], it is possible to support the hypothesis that trait mindfulness at work is able to sustain more authentic and true-selves values and behaviors. However, based on the COR theory [28] [29], the results of the present study improved previous research outcomes: mindfulness at work can accrue not only personal resources, but also job resources [61], such as the perception that teachers have of their own work as full of meaning. Moreover, this is consistent with JD-R assumption [19] [20], which states that one of the potential effects of personal resources is their impact on the way by which people perceive their work environment.

These results all together put more insights on the mechanisms that link mindfulness, as a stable personal resource adapted to the teaching context, to wellbeing outcomes. It could be confirmed that the re-perceiving process [10] favored by non-judgmental attitudes on the present-moment is able to sustain a less negative perception of potential threatening stimuli and a more meaningful view of the teaching work. The relevance of teaching mindfully is that it represents a fundamental resource that aids teachers to be more focused on the present moment of teaching activities and on students’ needs, favoring a more detached view of working problems that are not directly related to teaching tasks within the classroom. In this vein, being able to adopt a compassionate, open-hearted and affectionate orientation to present moment experiences during teaching, represents a fundamental self-regulatory resource that could strengthen the ability to invest in classroom relationships, management and instruction [62], and sustain rethinking about habitual stressful appraisal of working conditions, such as relations with students and colleagues.

Furthermore, the evidence that mindfulness could moderate a negative workload stress appraisal, but not the lack of job resources, is another important finding, which puts more insights on how this personal resource act within the theoretical model of the JD-R [19] [20]. On the one hand, it emerged that having higher levels of mindfulness acts as a protective factor against the presence of stress inducing factors such as the negative workload stress appraisal. In this vein it seems that teachers higher on mindfulness are more able to understand and control the activation of cognitive schemas that are based on the threatening perception of working events. On the other hand, the absence of a moderating effect between mindfulness and the meaningfulness of work could be explained by the assumption of the COR theory [28] [29] that burnout increases when people are lacking valued job resources. In this vein, it could be hypothesized that, for those teachers that experience low levels of meaningfulness of work, this could be not a valued job resource and even if teachers experience high levels of mindfulness, this personal resource is not able to compensate for the lack of meaningful work. Moreover, this result is consistent with the theoretical assumption about the main role of personal resources [30] within the JD-R model [19] [20], as they can primarily help to lessen the impact that job demands, or their negative stressful appraisal, have on the onset of burnout.

However, to date it is not so clear how job and personal resources can interact in predicting job burnout, and future research should invest more interest about the role exerted from mindfulness. In this vein, future studies could focus on the booster effect that the interaction between job resources and personal resources may have on work engagement [63].

Finally, these results give further insights on how mindfulness can act as a personal resource within the JD-R model, favoring a more in-depth view of its significance in the teaching profession, regarding which, except of the increasing interest paid to MBI’s effectiveness studies (e.g., [17]), no other studies have implemented correlational studies aimed at assess how trait mindfulness could be linked to occupational wellbeing outcomes.

### Limits and future directions

This study has some limits. First of all, results are not generalizable as the sample was composed of teachers of the primary, middle and secondary school in a limited metropolitan area in the north of Italy. Moreover, results should be affected from the common method variance bias, as we collected data only from one source of information. In this vein, future studies should collect longitudinal data considering, for example, daily fluctuations of mindfulness and related objectives physiological parameters. In this vein, results of the present study could be further analyzed within health promotion intervention aimed at sustain teachers’ wellbeing through MBI interventions. For example, future studies can improve the extant knowledge about the effect of mindfulness training [63] on mindfulness state, using diary studies to integrate the JD-R assumption about the potential mediating role of stress appraisal and the way by which teachers value their work as full of meaning.

Finally, it could be interesting, in a multilevel perspective, considering how mindfulness impacts on students’ engagement or classroom relational climate, or rating how students perceive the degree to which their teachers act in a mindful way. Future studies could examine changes in teachers’ mindfulness after MBIs.

## Supporting information

S1 File process. (SAV)

S2 File MPLUS. (DAT)
